# Antibiotic Resistance of Human Periodontal Pathogen *Parvimonas micra* Over 10 Years

**DOI:** 10.3390/antibiotics9100709

**Published:** 2020-10-17

**Authors:** Thomas E. Rams, Jacqueline D. Sautter, Arie J. van Winkelhoff

**Affiliations:** 1 Oral Microbiology Testing Service Laboratory, Department of Periodontology and Oral Implantology, Temple University School of Dentistry, Philadelphia, PA 19140, USA; jacqueline.sautter@temple.edu; 2 Department of Microbiology and Immunology, Temple University School of Medicine, Philadelphia, PA 19140, USA; 3 Department of Medical Microbiology, University Medical Center Groningen, University of Groningen, 9713 GZ Groningen, The Netherlands; ajwinkelhoff@laboral.nl; 4 Center for Dentistry and Oral Hygiene, University Medical Center Groningen, University of Groningen, 9713 GZ Groningen, The Netherlands

**Keywords:** anti-infective agents, periodontitis, drug resistance, in vitro, periodontal pocket, metronidazole, doxycycline, amoxicillin, clindamycin

## Abstract

Changes were evaluated over 10 years in the in vitro resistance of human periodontopathic strains of *Parvimonas micra* to four antibiotics. Subgingival biofilms culture positive for *P. micra* from 300 United States adults with severe periodontitis in 2006, and from a similar group of 300 patients in 2016, were plated onto anaerobically incubated enriched Brucella blood agar alone, or supplemented with either doxycycline (4 mg/L), clindamycin (4 mg/L), amoxicillin (8 mg/L), or metronidazole (16 mg/L). *P. micra* growth on antibiotic-supplemented media indicated in vitro resistance to the evaluated antibiotic concentration. *P. micra* resistance was significantly more frequent among patients in 2016, as compared to 2006, for doxycycline (11.3% vs. 0.3% patients; 37.7-fold increase), and clindamycin (47.3% vs. 2.0% patients; 23.7-fold increase) (both *p* < 0.001), whereas resistance to amoxicillin (2.3% vs. 1.0% patients) and metronidazole (0% vs. 0.3% patients) remained low and statistically unchanged between the two patient groups (*p*-values > 0.05). No *P. micra* isolates in 2006 or 2016 were jointly resistant in vitro to both amoxicillin and metronidazole. The alarming increases in subgingival *P. micra* resistance to doxycycline and clindamycin raise serious questions about the empiric use of these antibiotics, either locally or systemically, in the treatment of United States periodontitis patients harboring subgingival *P. micra*.

## 1. Introduction

Community-wide changes in subgingival bacterial composition and functional properties are considered important determinants of human periodontitis [1]. In addition, individual microbial species, such as *Parvimonas micra*, may play a key role in promoting subgingival bacterial dysbiosis, upregulating expression of periodontopathic virulence factors within the periodontal microbiome, and influencing periodontal treatment outcomes. *P. micra*, formerly known as *Peptostreptococcus micros* and *Micromonas micros* [2], is a Gram-positive, non-motile, anaerobic coccus widely recognized as a putative pathogen in human periodontitis [3]. *P. micra* is significantly more abundant in severe/moderate periodontitis patients than persons with periodontal health, gingivitis, and/or mild periodontitis [1,4]. The organism is recognized as a member of the human periodontitis core microbiome [1], and is included in the orange complex cluster group of subgingival bacteria significantly associated with deep periodontal pockets [5]. Pre-treatment presence of subgingival *P. micra* in periodontitis patients is a significant predictor of post-treatment persistence of residual ≥5 mm periodontal pockets that bleed on probing [6]. Additionally, elevated subgingival levels of *P. micra* remaining after conventional mechanical-surgical treatment of severe periodontitis patients predisposes them to a significantly increased risk of further periodontal breakdown [7,8,9]. *P. micra* is proposed to act as a “keystone” pathogen in the development of a dysbiotic subgingival microbial community and onset of periodontitis in some patient populations [10]. In regard to this, a *P. micra*-derived signaling molecule enhances growth and virulence of the major periodontal pathogen *Porphyromonas gingivalis* by stimulating a 13-fold greater expression of *P. gingivalis* proteolytic gingipain enzymes [11].

*P. micra* is often poorly removed from periodontitis sites with conventional forms of periodontal therapy, such as repeated root surface debridement, periodontal access flap surgery, systematic maintenance care, and meticulous supragingival plaque control [6,7,8,9,12]. This may be due to marked adherence of *P. micra* to gingival crevicular epithelium [13], which is less likely to be affected by periodontal root instrumentation than tooth-associated microbial biofilms. As a result, *P. micra* is frequently recovered from refractory periodontitis patients [7,8,14,15], for whom systemic antibiotic regimens are often prescribed [16]. *P. micra* is better removed from the subgingival microbiota of periodontitis patients treated with supplemental systemic antibiotic therapy than with mechanically-based periodontal therapy alone [6,17,18].

Little data of recent origin is available on the antibiotic susceptibility of *P. micra* in human periodontitis lesions. Studies carried out nearly 30 years ago found *P. micra* in United States periodontitis patients to be highly susceptible in vitro to penicillin, clindamycin, and metronidazole, with 90% of tested strains inhibited by 0.25, 1.0, and 4.0 mg/L of the antibiotics, respectively [7]. Similar findings reported over the next dozen years found no or relatively little in vitro antibiotic resistance among subgingival *P. micra* from periodontitis patients in the United States [19,20,21], Germany [22,23], Spain [24,25], and the Netherlands [24,25]. Exceptions to this were in France, where 40% of tested *P. micra* clinical isolates were resistant in vitro to metronidazole [26], and in Brazil, where 84% of *P. micra*-positive sites in untreated periodontitis patients harbored strains resistant in vitro to 4 mg/L of tetracycline [27]. A later study involving 364 *P. micra*-positive severe periodontitis patients in the United States evaluated prior to 2010 found in vitro resistance of the organism to be negligible to metronidazole and amoxicillin (only 0.6% patients each), rare to clindamycin (1.4% patients), and more frequent to doxycycline (18.4% patients) [28]. In comparison, a 2012 Dutch study of 50 *P. micra*-positive periodontitis patients found no or little in vitro resistance to metronidazole, amoxicillin, amoxicillin/clavulanic acid, clindamycin, azithromycin, or tetracycline [29].

Global antibiotic surveillance programs have revealed the progressive emergence of antibiotic resistance among bacteria in life-threatening medical infections [30], including anaerobic cocci such as *P. micra* [31], with increased morbidity and mortality rates reported in patients infected with antibiotic-resistant bacterial strains [30]. The World Health Organization and the United Nations have declared the rapid rise of microbial antibiotic resistance “a global crisis that threatens a century of progress in health” [32]. At present, no data is available evaluating possible changes over time in the antibiotic sensitivity profile of periodontal *P. micra*. Thus, it is not known if the progressive temporal increase in antibiotic resistance among medically-important bacteria is also taking place in periodontal pockets with *P. micra*. To help address this issue, this study evaluated, among United States adults with severe periodontitis, changes over a 10 year period in the prevalence of in vitro resistance of subgingival *P. micra* to four antibiotics commonly recommended for use in human periodontal disease therapy.

## 2. Materials and Methods

### 2.1. Nature of Study

This study involved a retrospective analysis, after removal of all unique patient identifiers, of pre-existing clinical and microbiological data archived at the Oral Microbiology Testing Service (OMTS) Laboratory, Temple University School of Dentistry, Philadelphia, PA, USA. The OMTS Laboratory is licensed for high complexity bacteriology and bacterial susceptibility analysis by the Pennsylvania Department of Health, and is federally certified by the United States Centers for Medicare and Medicaid Services to be in compliance with Clinical Laboratory Improvement Amendment (CLIA) regulations covering proficiency testing, quality control, patient test management, personnel requirements, and quality assurance standards required of clinical laboratories engaged in diagnostic testing of human specimens in the United States.

### 2.2. Patients

A total of 300 consecutive adults (145 males and 155 females; aged 36 to 82 years; mean age ± standard deviation (SD): 54.7 ± 10.6 years; 24.0% current smokers) found to be culture positive for subgingival *P. micra* in 2006, and 300 consecutive adults (148 males and 152 females; aged 35 to 85 years; mean age ± SD: 56.4 ± 10.9 years; 14.3% current smokers) found to be culture positive for subgingival *P. micra* during 2016 were identified in the archived records of the OMTS Laboratory and included in the present study. They were diagnosed with untreated severe periodontitis (equivalent to localized to generalized Stage III/Grade B or C periodontitis [33]) by periodontists in private dental practices in the United States, and presented with at least three periodontal sites with ≥6 mm probing depths. Samples from patients less than 35 years of age, diagnosed with molar/incisor pattern forms of periodontitis, or reporting antibiotic use within six months before sampling were excluded. The subgingival samples were processed in 2006 and 2016 using the same standardized laboratory protocol and laboratory personnel, who were masked to the clinical status of the patients and their inclusion in the present analysis. Approval for this secondary data analysis was granted by the Temple University Human Subjects Institutional Review Board (protocol #24458).

### 2.3. P. micra Culture and Identification

Subgingival biofilms were sampled from each study patient by their diagnosing periodontist with sterile paper points from three to five periodontal sites exhibiting deep periodontal probing depths (>6 mm) and bleeding on probing, following standardized sampling procedures previously described [28]. The subgingival specimens were pooled together per patient into a glass vial containing anaerobically prepared and stored viability medium Gothenburg anaerobic (VMGA) III transport medium [34], and delivered within 24 h to the OMTS Laboratory. Serial 10-fold dilutions of the microbial samples were plated onto enriched Brucella blood agar (EBBA) primary isolation plates [28], and incubated at 37 °C for seven days in jars containing an 85% N_2_-10% H_2_-5% CO_2_ anaerobic atmosphere introduced by an automatic jar evacuation and replacement system (Anoxomat Mark II, Advanced Instruments, Inc., Norwood, MA, USA).

Total cultivable anaerobic viable counts and *P. micra* colony-forming units (CFU) were enumerated on incubated EBBA primary isolation plates. *P. micra* was identified as forming small (minute to 1.0 mm in diameter), shiny, non-hemolytic, catalase-negative, opaque white, circular, convex, dome-shaped surface colonies comprised of Gram-positive, non-motile, anaerobic cocci [7]. Selected clinical isolates phenotypically identified as *P. micra* at several time points in 2016 were subjected to matrix-assisted laser desorption/ionization time-of-flight (MALDI-TOF) mass spectrometry testing, using methods and criteria previously described [35,36], as part of the OMTS Laboratory’s routine quality assurance and proficiency testing protocols, to confirm *P. micra* identification. Proportional patient recovery of *P. micra* isolates per subgingival specimen were calculated as the percent recovery of *P. micra* CFU among total cultivable anaerobic viable counts as determined on non-antibiotic supplemented EBBA plates.

### 2.4. In Vitro Antibiotic Resistance Testing

Subgingival sample aliquots were also inoculated onto EBBA culture plates supplemented with non-susceptible breakpoint concentrations of either 4 mg/L of doxycycline or clindamycin, 8 mg/L of amoxicillin, or 16 mg/L of metronidazole (all antibiotics obtained as pure powder from Sigma-Aldrich, St. Louis, MO, USA), followed by anaerobic incubation at 37 °C for seven days. *P. micra* growth on antibiotic-supplemented media indicated in vitro resistance to the evaluated antibiotic concentration. The designated antibiotic breakpoint concentration values were previously used in periodontal and peri-implant microbiology antibiotic resistance studies [28,37,38,39], and represent non-susceptible/resistant breakpoint concentrations against anaerobic bacteria for amoxicillin, clindamycin, and metronidazole as recommended by the Clinical and Laboratory Standards Institute (CLSI) [40], and for doxycycline as recommended by the French Society for Microbiology [41]. Incorporation of non-susceptible antibiotic breakpoint concentrations into primary isolation culture plates has excellent correlation (*r*^2^ = 0.99) with the CLSI-approved agar dilution susceptibility assay for identification of antibiotic-resistant periodontal microorganisms [20]. *Bacteroides thetaiotaomicron* ATCC 29741, *Clostridium perfringens* ATCC 13124, and a multi-antibiotic-resistant clinical periodontal isolate of *Fusobacterium nucleatum* were used as positive and negative quality controls for antibiotic resistance testing on antibiotic-supplemented EBBA plates.

### 2.5. Data Analysis

Descriptive analysis tabulated study patient demographic and behavioral characteristics, and the mean proportional cultivable *P. micra* recovery per patient from non-antibiotic-containing EBBA plates. For each of the test antibiotics, the number and proportion of patients positive with antibiotic-resistant strains of subgingival *P. micra* was determined, along with the mean proportional recovery of drug-resistant *P. micra* isolates per patient on various antibiotic-supplemented EBBA plates. A two-sample t-test compared differences between the 2006 and 2016 patient groups in mean age and mean cultivable levels of *P. micra* on non-antibiotic-supplemented EBBA plates. Fisher’s exact test examined differences between the two patient groups in the proportion of males, current smokers, and patients with *P. micra* resistance to each of the test antibiotics. Logistic regression models evaluated, for each of the test antibiotics within each of the two patient groups, the relationship of age, gender, and smoking status to *P. micra* antibiotic resistance (the dependent variable coded as 0 = not detected in patient, and 1 = present in patient, with binary dummy codes assigned to categorical explanatory variables). A *p*-value ≤ 0.05 was required for all tests of statistical significance. The PC-based STATA/SE 16.1 for Windows (StataCorp PL, College Station, TX, USA) 64 bit statistical software package was used in the data analysis.

## 3. Results

### 3.1. Confirmation of P. micra Identification

A total of 12 clinical isolates phenotypically identified as *P. micra* in 2016 and evaluated with MALDI-TOF mass spectrometry analysis were all confirmed to be *P. micra*.

### 3.2. Patient Group Characteristics

No statistically significant differences in mean age or the proportion of males were found between the 2006 and 2016 patient groups (*p*-values > 0.05). However, current smokers were significantly more prevalent in the 2006 patient group (*p* = 0.004). *P. micra* averaged 12.3 ± 9.8% (SD) of the cultivable subgingival microbiota in severe periodontitis patients in 2006, and 10.9 ± 9.2% (SD) among severe periodontitis patients in 2016, which were not significantly different from each other (*p* = 0.072).

### 3.3. In Vitro Antibiotic Resistance Testing

Bacterial species employed as positive and negative quality controls (*B. thetaiotaomicron*, *C. perfringens*, and *F. nucleatum*) provided expected outcomes during in vitro antibiotic resistance testing.

Table 1 lists the prevalence of subgingival *P. micra* resistance to the four test antibiotics among the 2006 and 2016 patient groups.

Relatively little in vitro *P. micra* resistance to the test antibiotics was present in the 2006 patient group (Table 1). *P. micra* in vitro resistance was significantly more frequent in severe periodontitis patients in 2016, as compared to 2006, for doxycycline (11.3% vs. 0.3% patients; 37.7-fold increase), and clindamycin (47.3% vs. 2.0% patients; 23.7-fold increase) (both *p* < 0.001). Cultivable subgingival proportions of *P. micra* averaged 9.8% and 12.1% in 2016 patients positive with doxycycline and clindamycin-resistant strains, respectively (Table 1). In contrast, the prevalence of *P. micra* resistance remained low and statistically unchanged between patients tested in 2006 and 2016 for amoxicillin (2.3% vs. 1.0% patients) and metronidazole (0% vs. 0.3% patients) (both *p* > 0.05).

Joint in vitro resistance of *P. micra* to both doxycycline and clindamycin was significantly more frequent in 2016, with 11 (3.7%) positive patients, as compared to none in 2006 (*p* < 0.001). Two (0.7%) patients in 2016 yielded *P. micra* subgingival isolates jointly resistant to doxycycline, clindamycin, and amoxicillin, as compared to none in 2006. One (0.3%) patient in 2016 had *P. micra* resistance to both clindamycin and amoxicillin, as compared to none in 2006. None of the *P. micra* isolates in 2006 or 2016 were jointly resistant in vitro to both amoxicillin and metronidazole.

A total of 289 (96.3%) patients in 2006 did not reveal in vitro resistance to any of the four tested antibiotics among their *P. micra* subgingival isolates, whereas significantly fewer patients (132; 44.0%) in 2016 revealed a similar absence of *P. micra* antibiotic resistance (*p* < 0.001).

No statistically significant associations were found with logistic regression analysis between *P. micra* antibiotic resistance and patient age, gender, and smoking status for any of the test antibiotics within each of the two patient groups.

## 4. Discussion

This study provides the first United States patient data in 25 years on possible changes over time in the antibiotic resistance profile of a periodontal bacterial pathogen. The major finding is that marked increases occurred over a 10 year period from 2006 to 2016 in the in vitro resistance of fresh clinical isolates of periodontal *P. micra* to doxycycline and clindamycin, but not to amoxicillin or metronidazole. Particularly alarming were the 37.7-fold (3767%) and 23.7-fold (2365%) increases in resistance to doxycycline and clindamycin, respectively, which were detected in subgingival *P. micra* clinical isolates tested in 2016, as compared to those evaluated in 2006. In contrast, no significant 10 year changes from 2006 to 2016 were found with the low to negligible in vitro resistance of periodontal *P. micra* to amoxicillin and metronidazole.

These data are consistent with comparisons made in United States periodontal patients between 1991–1995 and 1980–1985 in the prevalence of antibiotic-resistant periodontal microorganisms [42]. Statistically significant increases were found during the period 1991–1995 in the percentage of periodontal species resistant to tetracycline, doxycycline, and amoxicillin, but not to clindamycin or erythromycin, as compared to 1980–1985 [42]. The present study, conducted more than a generation later with periodontal *P. micra*, indicates that microbial resistance to tetracycline family antibiotics continues to progressively rise in the human subgingival microbiome, and that marked increases in *P. micra* drug resistance are now also found with clindamycin. The emergence of joint drug resistance to both doxycycline and clindamycin among periodontal *P. micra* strains in 2016 (3.7% of patients), as compared to none in 2006, is also of concern.

These findings have potentially important clinical patient care implications. Since metronidazole was consistently effective in vitro against periodontal *P. micra*, systemic administration of metronidazole alone, or in combination with amoxicillin, is thus appropriate to consider for *P. micra*-infected periodontitis patients as an adjunct to conventional mechanical root debridement therapy, in accordance with American Dental Association clinical practice guidelines published in 2015 [43]. Importantly, no *P. micra* clinical isolates in either 2006 or 2016 were jointly resistant in vitro to both metronidazole and amoxicillin, consistent with the marked in vivo subgingival suppression of *P. micra* exerted by joint systemic metronidazole-amoxicillin periodontitis drug therapy [6,17,18], which is increasingly employed in clinical periodontal practice [16,43]. 

In contrast, clindamycin and doxycycline, which in past years provided benefit for some refractory periodontitis patients [16], appear increasingly more limited as potential therapeutic agents against subgingival *P. micra*, due to increasing *P. micra* drug resistance, and as adjunctive antimicrobial agents in periodontal disease treatment. Administration of systemic doxycycline to severe periodontitis patients colonized by doxycycline-resistant subgingival *P. micra* results in undesirable post-treatment increases in the prevalence of drug-resistant strains [21]. Placement of sustained-release devices eluting doxycycline from gels [44], or minocycline from microspheres [45], into periodontal pockets harboring doxycycline-resistant *P. micra* strains may similarly induce adverse microbiological shifts leading to clinical therapeutic failure, and potentially accelerate spread of antimicrobial resistance within the human microbiome. Due to the frequent presence of *P. micra* in periodontitis lesions [1,4], and its increasing resistance to doxycycline and clindamycin (Table 1), empiric administration of these two drugs in human periodontal disease therapy should be discouraged. Instead, the use of antibiotics in periodontal practice should ideally be based upon microbiological analysis of subgingival biofilms and the results of in vitro antibiotic susceptibility testing [3], in addition to consideration of the patient’s clinical status, medical history, and other medications [16].

The present study data are limited in several aspects. Different patients comprised the two study groups, with no longitudinal cohort of periodontitis patients evaluated during the 10 year study period. Additional demographic, clinical, microbiologic, genetic, and/or behavioral differences not evaluated within the two patient groups may have influenced (confounded) the study findings. The study patients may not be statistically representative of periodontitis patients throughout the United States. No clinical or radiographic evaluations of the study patients were made by calibrated examiners, independent of the private practice periodontists who collected and submitted diagnostic information on the patients. However, their identification in each study patient of three or more periodontal pockets with ≥6 mm probing depths strongly correlates (94.1% positive predictive value) with detection of severe periodontal attachment loss in adults [46]. The long-term history of antibiotic use among the study patients was not available. Only subgingival *P. micra* clinical isolates were evaluated in this report, without analysis of other subgingival species which may possess differing in vitro antibiotic susceptibility profiles. Minimal inhibitory concentration values of the test antibiotics against *P. micra* clinical isolates were not determined in this study, and antibiotic resistance genes in the *P. micra* strains were not identified.

## 5. Conclusions

Marked and alarming increases were found in 2016, as compared to 2006, in the prevalence of fresh clinical isolates of subgingival *P. micra* resistant to doxycycline and clindamycin, but not to amoxicillin or metronidazole, among severe periodontitis patients in the United States. These findings raise serious questions about the empiric use of doxycycline or clindamycin, either locally or systemically, in the treatment of periodontitis patients in the United States harboring subgingival *P. micra*.

## Figures and Tables

**Table 1:** Patients with subgingival *P. micra* resistant in vitro to antibiotic breakpoint concentrations.

Year (*n*,* % Recovery ^†^)	Clindamycin (4 mg/L) ^‡^	Doxycycline (4 mg/L) ^‡^	Amoxicillin (8 mg/L) ^‡^	Metronidazole (16 mg/L) ^‡^
**2006:**				
*n*	6 (2.0)	1 (0.3)	3 (1.0)	1 (0.3)
% recovery	16.2 ± 5.0	22.2	9.1 ± 2.5	13.5
**2016:**				
*n*	142 (47.3) ^§^	34 (11.3) ^§^	7 (2.3)	0
% recovery	12.1 ± 10.4	9.8 ± 6.6	8.5 ± 8.3	0

* Number (%) of patients with periodontal *P. micra* resistant in vitro to antibiotic breakpoint concentration; ^†^ mean ± SD percentage recovery of antibiotic-resistant *P. micra* in positive patients; ^‡^ Antibiotic breakpoint concentration incorporated into EBBA culture plates; ^§^ *p* < 0.001 compared to 2006.
